# Annual of the Universal Medical Sciences

**Published:** 1888-12

**Authors:** 


### Book Reviews.

ANNUAL OF THE UNIVERSAL MEDICAL SCIENCES. A YEARLY REPORT OF THE PROGRESS OF THE GENERAL SANITARY SCIENCES THROUGHOUT THE WORLD. Edited by Charles E. Sajous, M. D., Lecturer on Laryngology and Rhinology in Jefferson Medical College, Philadelphia, etc., and seventy associate editors, assisted by over two hundred corresponding editors, collaborators and correspondents. Illustrated with chromos, lithographs, engravings and maps. Volumes I, II, III, IV, V, 1888. Philadelphia and London: F. A. Davis, publisher.

In the editor's preface we learn that the object of the Annual of the Universal Medical Sciences is to collate the progressive features of medical literature at large, and clinical data from countries in which no literature exists, and to present the whole once a year in a continued form, prepared by writers of known ability.

The vast proportions of this undertaking should lead the profession to anticipate a general rather than a special notice of the various topics included under the seventy headings of the work, and after a careful examination of the contents of these 2,713 pages, it is found that many subjects have not received that attention which their importance would have warranted. The space allowed each subject does not suffice for the many details in regard to points which are essential to a proper comprehension, and it is evident that rare abnormalities have been collected by writers in preference to giving minutiæ of cases of more practical importance. The editor might have met the wants of medical men more satisfactorily by presenting a complete exposition of a less number of topics.

Although there are five volumes, the large type and leaded lines afford only 400 words to the page, and the five contain less than one volume of another record of the medical sciences, containing 812 pages with 1,400 words to the page, which is in course of publication at present. It thus becomes patent that it was simply impossible to do justice to the matters treated of; and though the work is gotten up in very good style, with illustrations in the highest order of excellence, it falls short of what the profession had a right to expect from such an array of talent as the editor has brought to his aid in its preparation.

While there is much valuable information contained in these elegant pages, the close observer cannot fail to note that some erroneous statements which have appeared in the medical journals of this country have not been corrected by comparing them with the original publications abroad. In the future issues of this annual it is believed that the defects referred to may be remedied, and that it may fill the measure of the expectations of its numerous readers and subscribers throughout this country.

